# The Fate of Two Unstoppable Trains After Arriving Destination: Replisome Disassembly During DNA Replication Termination

**DOI:** 10.3389/fcell.2021.658003

**Published:** 2021-07-21

**Authors:** Yisui Xia

**Affiliations:** The MRC Protein Phosphorylation and Ubiquitylation Unit, School of Life Sciences, University of Dundee, Dundee, United Kingdom

**Keywords:** p97/CDC48/VCP, SCFcpsdummyDia2, CUL2cpsdummyLRR1, CMG, ubiquitylation, replisome disassembly

## Abstract

In eukaryotes, the perfect duplication of the chromosomes is executed by a dynamic molecular machine called the replisome. As a key step to finishing DNA replication, replisome disassembly is triggered by ubiquitylation of the MCM7 subunit of the helicase complex CMG. Afterwards, the CDC48/p97 “unfoldase” is recruited to the ubiquitylated helicase to unfold MCM7 and disassemble the replisome. Here we summarise recently discovered mechanisms of replisome disassembly that are likely to be broadly conserved in eukaryotes. We also discuss two crucial questions that remain to be explored further in the future. Firstly, how is CMG ubiquitylation repressed by the replication fork throughout elongation? Secondly, what is the biological significance of replisome disassembly and what are the consequences of failing to ubiquitylate and disassemble the CMG helicase?

## Overview of DNA Replication Termination

The accurate and complete duplication of the chromosomes is essential for the inheritance of genetic information. It is initiated during the S-phase of the cell cycle by the assembly of a pair of bi-directional replisome complexes at many origins of DNA replication, followed by semi-conservative DNA synthesis until each chromosome is perfectly duplicated (O’Donnell et al., 2013; Bell and Labib, 2016). Every beginning has an end, so as DNA replication. Its final processes define its termination. When two replication forks from neighbouring origins converge with each other, replication terminates and the remaining stretch of parental DNA between the two replisomes is unwound. Subsequently, a single-stranded gap exists between the 3′ end of the leading strand of one fork and the downstream Okazaki fragment of the opposing fork. This gap is then filled and the Okazaki fragment processed, leading to completion of DNA synthesis for a given replicon. The replicated sister chromatids are still linked by catenanes that are resolved subsequently by topoisomerase II, which is important to ensure that chromosome segregation can proceed successfully during mitosis (Dewar et al., 2015; Dewar and Walter, 2017).

The key regulated step during DNA replication termination is the disassembly of the replisome. In eukaryotes, the replisome is a large multi-protein super-complex (Baretic et al., 2020). The core of the replisome is the replicative helicase known as CMG (Cdc45-MCM-GINS), which is formed by the six Mcm2-7 ATPases, the Cdc45 protein, and the GINS complex. CMG encircles the leading strand template DNA strand and functions as a 3′–5′ DNA helicase during replication fork progression (Eickhoff et al., 2019; Yuan et al., 2020). It is important that CMG remains tightly associated with replication forks throughout replication elongation, since the Mcm2-7 catalytic core can only be loaded around DNA during G1-phase. The remarkably stable association of CMG with replication forks implies that an active mechanism is required to disassemble the helicase and thus trigger replisome dissolution during DNA replication termination. Disassembly of CMG then leads to unloading of the associated replisome factors from chromatin, once DNA synthesis has been completed (Maric et al., 2014; Moreno et al., 2014).

CMG disassembly is initiated by ubiquitylation of its Mcm7 subunit, which then is rapidly unfolded by the Cdc48/p97 AAA + ATPase in association with its major adaptors Npl4 and Ufd1 (Deegan et al., 2020). In this review, we focus on the mechanism of CMG disassembly during eukaryotic DNA replication termination.

## Fork Convergence

Except at chromosome ends or at nicks or breaks in the DNA template, fork convergence is an essential pre-requisite for replisome disassembly during termination. During fork progression, DNA unwinding by the replicative helicase causes torsional strain that induces positive supercoils ahead of each replisome. Type I or II topoisomerases remove these supercoils and are essential for continued fork progression in both prokaryotes and eukaryotes. However, when two forks converge and are less than ∼150 bp apart, there is no longer space to form DNA supercoils ahead of the two replisomes. Under these conditions, the topological stress that results from unwinding the final stretch of parental DNA is resolved by replisome rotation, which leads to precatenane formation behind the converging replisomes. In bacteria and viruses, the removal of precatenanes by type II topoisomerases is crucial to allow converging replisomes to unwind the final stretch of parental DNA (Dewar and Walter, 2017). However, depletion of type II topoisomerase does not block fork convergence in budding yeast cells (Baxter and Diffley, 2008), although Top2 does make a minor contribution to the efficiency of fork convergence when replication terminates in a reconstituted DNA replication system (Deegan et al., 2019; Devbhandari and Remus, 2020). A similar result was reported in egg extracts of *Xenopus laevis*, in which Top2α promotes fork convergence by preventing the accumulation of topological stress from earlier stages of replication (Heintzman et al., 2019).

**FIGURE 1:**
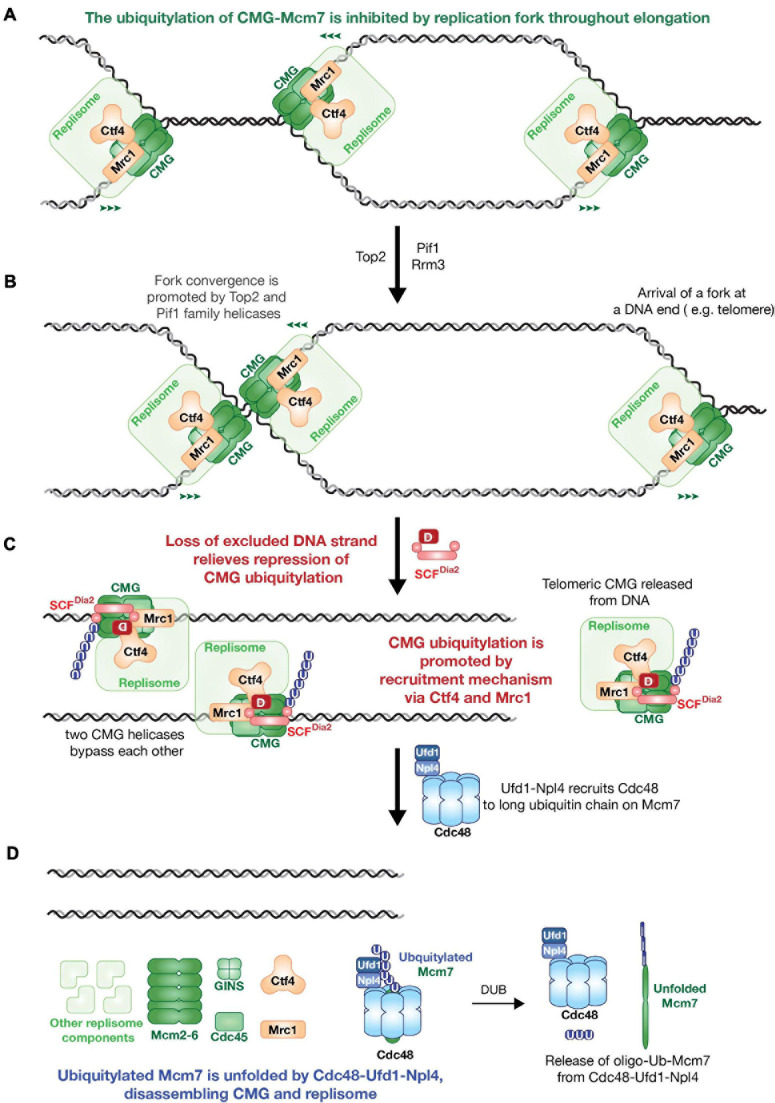
The model of *Saccharomyces cerevisiae* replisome disassembly during DNA replication termination. **(A)** CMG ubiquitylation is inhibited by replication fork during replication elongation. **(B)** Fork convergence is promoted by type II topoisomerases and Pif1 family helicases. **(C)** Loss of excluded strand relieves repression of CMG ubiquitylation, then SCF^Dia2^ dependent Mcm7 ubiquitylation is promoted by recruitment mechanism via replisome components Ctf4 and Mrc1. **(D)** Ufd1-Npl4 recruits Cdc48 to ubiquitylated CMG to unfold poly-ubiquitylated Mcm7 subunit and disassemble replisome.

The fact that *Saccharomyces cerevisiae* DNA replication can be reconstituted with purified proteins *in vitro* gives a unique opportunity to study the molecular mechanism of termination (Yeeles et al., 2015, 2017). In reactions containing all the factors that are essential for initiation and elongation, converging replication forks were seen to stall even in the presence of topoisomerases (Deegan et al., 2019). This observation led to the identification of a role for the Pif1 family of DNA helicases during DNA replication termination (Figure 1A). Budding yeast Pif1 and Rrm3 are monomeric DNA helicases that have low processivity and 5′–3′ unwinding polarity. Both Pif1 and Rrm3 can support termination in the reconstituted budding yeast DNA replication system, in contrast to other 5′–3′ helicases including a bacterial Pif1 orthologue that is highly active as a helicase *in vitro* (Deegan et al., 2019). These findings suggest that budding yeast Pif1/Rrm3 might rely on specific interactions with the yeast replisome, though this remains to be determined.

*In vivo* studies have also implicated the single fission yeast Pif1 helicase (Pfh1) in the convergence of DNA replication forks during termination (Steinacher et al., 2012). The situation in metazoa is likely to be more complicated, given the greater number of 5′–3′ DNA helicases in metazoan species. It will be interesting in future studies to investigate whether other such helicases or other uncovered pathways can also promote fork convergence.

## CMG Disassembly in Budding Yeast

**FIGURE 2:**
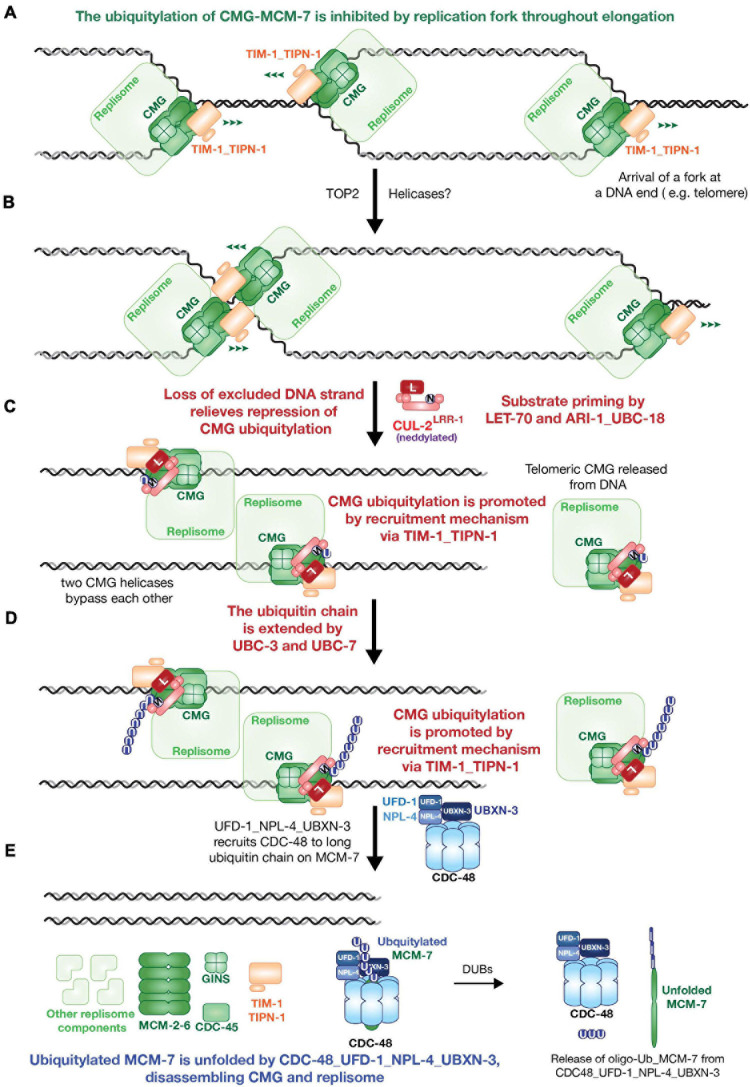
A possible model of *Caenorhabditis elegans* replisome disassembly during DNA replication termination. **(A)** Replication fork structure blocks CMG ubiquitylation during replication elongation. **(B)** Fork convergence is supported by type II topoisomerases or other uncovered pathways **(C)** CMG ubiquitylation starts with substrate priming step via LET-70 or ARI-1_UBC-18 with neddylated CUL-2^LRR–1^ when the repression from fork DNA is eliminated. **(D)** E2s UBC-3 or UBC-7 work with neddylated CUL-2^LRR–1^ to extend ubiquitin chain on MCM-7. Both of substrate priming and ubiquitin chain extension are stimulated by recruitment of CUL-2^LRR–1^ via TIM-1_TIPN-1. **(E)** UFD-1_NPL-4_UBXN-3 recruits CDC-48 to ubiquitylated CMG, as a result, MCM-7 is unfolded and replisome is disassembled.

CMG ubiquitylation is blocked when converging replication fork arrest in the absence of Pif1-family DNA helicases, indicating that the helicase is normally only ubiquitylated after DNA synthesis has been completed (Figures 1A,B; Deegan et al., 2020). The trigger for CMG-Mcm7 ubiquitylation during DNA replication termination is still understood poorly. Inhibiting CMG ubiquitylation throughout elongation is likely to be important for the preservation of genome integrity, otherwise forks could become permanently arrested. A recent study in *S. cerevisiae* and *X. laevis* egg extract suggested that CMG ubiquitylation is inhibited throughout elongation by the Y-shaped DNA structure of a replication fork, with which the helicase associates (Figures 1A, 2A; Deegan et al., 2020; Low et al., 2020). The mechanism of such inhibition remains to be determined.

Once CMG ubiquitylation is activated, a replisome associated E3 ubiquitin ligase drives the poly-ubiquitylation of CMG-Mcm7. The first such ligase to be characterised was the cullin 1-RING ligase in budding yeast known as SCF^Dia2^ (Maric et al., 2014). Ubiquitylation by as SCF^Dia2^ begins with the action of the E1 ubiquitin-activating enzyme known as Uba1, which transfers activated ubiquitin to the E2 ubiquiting conjugating enzyme Cdc34 (Deegan et al., 2020). In other species, the ubiquitin-like protein NEDD8 is essential for the function of cullin-RING ligase. The budding yeast NEDD8 orthologue Rub1 modifies the cullin scaffold as in other species, but is a non-essential protein. CMG ubiquitylation in *rub1Δ* cells was only reduced modestly, indicating that neddylation has little impact on the regulation of SCF^Dia2^ (Mukherjee and Labib, 2019).

SCF^Dia2^ is recruited to the CMG helicase by two components of budding yeast replisome, known as Ctf4 and Mrc1 (Morohashi et al., 2009; Maculins et al., 2015). These two factors jointly ensure the very high efficiency of CMG ubiquitylation by SCF^Dia2^, thereby pushing ubiquitylated Mcm7 over a ubiquitin threshold that governs the action of the Cdc48/p97 ATPase (Figure 1C; Deegan et al., 2020). Cdc48/p97 ATPase is a hexameric ATPase that disrupts protein structure and transports unfolded polypeptides through its central channel (Bodnar and Rapoport, 2017; Cooney et al., 2019; Twomey et al., 2019). In budding yeast, Cdc48 is recruited to ubiquitylated substrates by its Ufd1-Npl4 cofactors. A combination of biochemical and structural studies have shown that Cdc48-Ufd1-Npl4 can only unfold proteins that are conjugated to at least five ubiquitins (Twomey et al., 2019; Deegan et al., 2020). The replisome-recruitment mechanism of SCF^Dia2^ guarantees that CMG disassembly is highly efficient during DNA replication termination. In addition, the requirement of Cdc48 for long ubiquitin chains on its substrates provides a form of quality control. Unscheduled ubiquitylation events during replication elongation are likely to be inefficient and would only produce short ubiquitin chains, thereby preventing premature Cdc48-Ufd1-Npl4 dependent CMG disassembly (Figure 1D; Deegan et al., 2020).

The mechanism by which Cdc48-Ufd1-Npl4 disassembles ubiquitylated CMG involves the specific unfolding of the ubiquitylated Mcm7 subunit (Deegan et al., 2020), likely initiated by unfolding of one of the ubiquitin moieties conjugated to Mcm7 (Twomey et al., 2019). Unfolding of Mcm7 collapses CMG and leads to disassembly of the replisome. The fate of unfolded Mcm7 is probably to be degraded by proteasome (Figure 1D; Deegan et al., 2020).

## CMG Disassembly in Metazoa

Studies of CMG disassembly in *Caenorhabditis elegans* and *X. laevis* have shown that CMG is ubiquitylated on its MCM7 subunit as in budding yeast, and disassembled by the CDC48/p97 ATPase (Dewar et al., 2017; Sonneville et al., 2017). However, orthologues of the F-box protein Dia2 are not apparent in metazoa and studies of *C. elegans* early embryos, *X. laevis* egg extracts, and mouse ES cells showed that a different E3 ligase known as CUL-2^LRR–1^ drives the poly-ubiquitylation of MCM7 during DNA replication termination (Figures 2C,D; Dewar et al., 2017; Sonneville et al., 2017; Villa et al., 2021). Work with *X. laevis* egg extracts showed that CUL2^LRR1^ is only recruited to the replisome during replication termination. A very recent study indicates that recruitment of CUL2^LRR1^ is blocked throughout elongation by the association of CMG with the DNA replication fork (Dewar et al., 2017), mirroring the regulation of yeast SCF^Dia2^.

The action of cullin-RING ubiquitin ligases is more complex in metazoa than in budding yeast. Work with human cells showed that cullin-RING ligases are activated by neddylation of the cullin subunit (Baek et al., 2020). Furthermore, the first ubiquitin is conjugated to substrates of metazoan cullin-RING ligases by different enzymes to those that subsequently elongate the K48-linked ubiquitin chain. Such priming of ubiquitylation can occur in two different ways. Firstly, the RING subunit of the cullin-RING ligase can activate the E2 UBE2D (UBCH5) to add the first ubiquitin to the substrate. Alternatively, a “RING between RING” or RBR ligase of the Ariadne family can associate with neddylated cullin scaffold and receive activated ubiquitin from a cognate E2, before transferring ubiquitin to substrate (Scott et al., 2016; Baek et al., 2020). Subsequently, the E2 enzymes UBE2R1/2/CDC34 and UBE2G1 function redundantly to elongate a K48-linked ubiquitin chain (Hill et al., 2019).

CMG ubiquitylation in *Xenopus* egg extracts is inhibited by MLN4924, a specific inhibitor of the E1 enzyme for the NEDD8 pathway (Dewar et al., 2015). However, until recently the ubiquitylation of metazoan CMG had not been reconstituted *in vitro*, and the enzymes responsible for ubiquitin priming and elongation on CMG had not been characterised in any metazoan species. However, *C. elegans* CMG ubiquitylation was recently reconstituted with a set of purified proteins (Xia et al., 2021). Orthologues of human UBCH5 and the Ariandne ligase ARIH1 cooperate redundantly with neddylated CUL-2^LRR–1^ to prime ubiquitylation of CMG-MCM-7 (Figure 2C), as predicted by studies of human cullin-RING ligases. Moreover, a ubiquitin-chain on primed CMG-MCM-7 is then extended redundantly by two E2s, UBC-3 (nematode orthologue of human CDC34/UBC2R) and UBC-7 (orthologue of human UBE2G1) (Figure 2D).

Interestingly, the replisome components TIM-1_TIPN-1, whose orthologues are TIMELESS-TIPIN in mammalian cells, were found to recruit CUL-2^LRR–1^ to worm CMG (Xia et al., 2021). Depletion of TIM-1_TIPN-1 dramatically compromises the ubiquitylation of MCM-7 both *in vitro* and *in vivo* (Figures 2C,D). These findings indicate that the metazoan and yeast replisomes use different but analogous recruitment mechanisms for CUL-2^LRR–1^ and SCF^Dia2^, in order to push CMG-MCM-7 ubiquitylation over the ubiquitin threshold of the CDC48/p97 ATPase.

In addition to UFD1-NPL4, metazoan cells contain a range of other partners of CDC48/p97 that are thought to recruit the unfoldase to specific targets or particular subcellular locations. In *C. elegans*, together with CDC-48_UFD-1_NPL-4, an adaptor protein known as UBXN-3 (the nematode orthologue of human FAF1) was shown to be required for a second pathway of CMG disassembly in *C. elegans* early embryos that acts during mitosis (Sonneville et al., 2017), in order to process sites of incomplete DNA replication (Sonneville et al., 2019). Recent work showed that UBXN-3 is also critical for CMG helicase unloading during DNA replication termination, in worms depleted for TIM-1_TIPN-1 (Xia et al., 2021). Moreover, UBXN-3 stimulates the ability of CDC-48_UFD-1_NPL-4 to disassemble poly-ubiquitylated CMG *in vitro* (Figure 2E), revealing a more complicated mechanism for the metazoan CMG disassembly machinery. It remains to be determined why metazoan cell need an additional adaptor in order to disassemble ubiquitylated CMG, especially when p97-UFD1-NPL4 is able to unfold a model ubiquitylated protein *in vitro* (Pan et al., 2021). The unfolding mechanism for ubiquitylated MCM-7 is probably similar in metazoa to that observed in yeast, but greater insight is likely to come from structural studies in the future.

## Perspective

It is now clear that the termination of eukaryotic DNA replication is regulated just as carefully as the initiation of DNA synthesis. Replisome disassembly is the key regulated step during termination and although the principal steps have now been identified, several critical questions await further investigation.

The first question is in regard to the beginning of this process. What is the signal of MCM7 ubiquitylation? As discussed above, a series of observations with yeast and *Xenopus* egg extracts indicate that CMG ubiquitylation is inhibited throughout elongation by the DNA structure of a replication fork. It means the termination of DNA replication removes the Y-shaped DNA structure of a fork, thereby exposing CMG to ubiquitylation by SCF^Dia2^ in budding yeast or CUL2^LRR1^ in metazoa. The same mechanism would apply when two forks converge and when a single fork arrives at a DNA end (Deegan et al., 2020; Low et al., 2020). However, the mechanism by which the Y-shaped fork DNA structure represses CMG ubiquitylation still remains unclear. The timing of recruitment of SCF^Dia2^ to the yeast replisome is not known, but CUL2^LRR1^ isn’t recruited at stall fork but terminated fork in *X. laevis* egg extract (Dewar et al., 2017). Moreover, the E3 ligases are recruited by members of replisome components to ensure efficient ubiquitylation in yeast and nematode (Deegan et al., 2020; Xia et al., 2021), which suggests the possibility that this kind of recruitment of E3 ligase is inhibited by repressive fork DNA structure. Furthermore, there is another possibility that ubiquitylation sites on MCM7 are protected by fork DNA structure. During unwinding, CMG encircles and translocates along the leading strand while excluding the lagging strand template. It is still difficult to interpret how the flexible lagging strand can prevent the lysine sites located at the N-terminal of MCM7 from being ubiquitylated. It may suggest the possibility that the lagging strand machinery also contributes to protecting progressive replisome. In subsequent studies, structural biology will be important to determine how the yeast and metazoan E3 ligases engage with the replisome and gain access to their cognate ubiquitylation sites on MCM7.

Another important question regards the physiological importance of replisome disassembly during DNA replication termination. In budding yeast, Dia2 is a non-essential gene, but *dia2Δ* shows cold-sensitive growth defect and hyper-sensitive to the replication stress (Morohashi et al., 2009; Maculins et al., 2015). Similarly, LRR-1 is an essential gene that supports the normal growth in *C. elegans* (Ossareh-Nazari et al., 2016; Sonneville et al., 2017). In addition, FAF1, the orthologues of *C. elegans* UBXN-3 in human, is a tumour suppressor. These important genes’ being involved in CMG disassembly reveals the significance of this process. A clear S phase delay was observed in *lrr-1* depleted worm embryo (Sonneville et al., 2017) implies that the recycle of CMG subunits during S phase might be important to efficiently complete DNA synthesis. Although there is no evidence yet to confirm MCM7 is the primary substrate of SCF^Dia2^ or CUL2^LRR1^, the importance of CMG disassembly can be studied by generating MCM7 ubiquitylation sites mutated alleles in the future. Additionally, there is a back-up pathway of CMG ubiquitylation in metazoan. If CMG is failed to be disassembled during termination, MCM7 will be ubiquitylated by another RING E3 ligase TRAIP in mitosis (Deng et al., 2019). This mechanism increases the complexity of the significance of CUL2^LRR1^ dependent CMG ubiquitylation. The genomes fail to complete replication in S phase when the cells are challenged by replication stress. The regions of incompletely replicated DNA are processed in early mitosis via a process known as mitotic DNA repair synthesis (MiDAS; Minocherhomji et al., 2015). In this situation, the CMG cannot be disassembled by CUL2^LRR1^ for the incomplete DNA synthesis, as well as TRAIP is essential for MiDAS. It implies that the un-disassembled CMG complex becomes a potential barrier to the MiDAS pathway (Deng et al., 2019; Sonneville et al., 2019). It also provides a possibility that accumulated CMG complex could interfere with other processes on chromosomes. Moreover, TRAIP also leads to disassembly of post-termination CMG helicases in worms, frog egg extracts, and mammalian cells that lack the activity of CUL-2^LRR–1^ (Deng et al., 2019; Villa et al., 2021). It remains to be determined why TRAIP is unable to compensate for the lethal consequences of deleting the LRR-1 gene, both in *C. elegans* and also in mammalian cells where LRR1 is also an essential gene (from IMPC Viability Primary Screen [IMPC_VIA_001]).

The eukaryotic replisome is equivalent to an unstoppable train that proceeds inexorably throughout each replicon from initiation until DNA synthesis is finished. Subsequently, CMG disassembly removes the formerly unstoppable train from the track, upon arrival at the destination. Much still remains to be learnt about this highly complicated process, which appears to have diverged considerably during the course of eukaryotic evolution. The cullin-RING ubiquitin ligases SCF^Dia2^ and CUL2^LRR1^ are not found in other branches of eukarya such as plants, and it will be important to explore whether replisome disassembly during DNA replication termination follows universal principles in diverse eukaryotes, or else has evolved repeatedly and is not always dependent upon MCM7 ubiquitylation.

## Author Contributions

The author confirms being the sole contributor of this work and has approved it for publication.

## Conflict of Interest

The author declares that the research was conducted in the absence of any commercial or financial relationships that could be construed as a potential conflict of interest.
